# Prevalence of incidental findings in adult vs. adolescent patients in the course of orthodontic X-ray diagnostics

**DOI:** 10.1007/s00056-022-00399-2

**Published:** 2022-05-18

**Authors:** Daniela Klenke, Petra Santander, Charlotte Vehring, Anja Quast, Jan Sommerlath Sohns, Sebastian Krohn, Philipp Meyer-Marcotty

**Affiliations:** 1 Department of Orthodontics, University Medical Centre Göttingen, Göttingen, Germany; 2 Department of Nuclear Medicine, Hannover Medical School, Hannover, Germany; 3 Department of Prosthodontics, University Medical Centre Göttingen, Göttingen, Germany

**Keywords:** Orthopantomograms, Treatment planning, Lateral cephalogram, Orthodontic diagnostics, Age effect, Orthopantomogramme, Behandlungsplanung, Fernröntgenseitenbild, Kieferorthopädische Diagnostik, Alterseffekt

## Abstract

**Objective:**

Due to increasing numbers of adult patients presenting to orthodontic practices, an increase in incidental findings on diagnostic X‑rays, which are the cornerstone of orthodontic diagnostics, is expected. This raises the clinically relevant question of whether an age effect exists regarding prevalence, localisation and severity of incidental findings on orthodontic diagnostic X‑rays.

**Materials and methods:**

The clinical, primarily retrospective study examined pathological incidental findings from 600 orthopantomograms (OPT) and lateral cephalogram (LC) images in two groups of orthodontic patients (group I: 150 children/adolescents, age 11.89 ± 2.47 years; group II: 150 adults, age 27.03 ± 10.42 years). Prevalence, localisation and severity of the findings were recorded based on a classification sheet. The assessment was done by three experienced examiners following a systematic approach along the nine locations: mandible, maxilla, dentition, paranasal sinuses, temporomandibular joint, cranial base, orbit, cervical spine, soft tissues.

**Results:**

In all, 1458 incidental findings were detected, with 66% of the findings having occurred away from the dentition. There was a significant age effect (*p* < 0.001) with respect to the prevalence of incidental findings (group II—adults 1026 findings—OPT: 566/LC 460 vs. group I—children/adolescents 432 findings—OPT: 221/LC 211). Regarding localisation, incidental findings in adults commonly occurred in the dentition, paranasal sinuses and mandibular regions. Furthermore, analysis of the LC images revealed significantly more incidental findings in the area of the cranial base and cervical spine in adults (*p* < 0.001, *p* = 0.003). Categorisation according to the severity of the incidental findings showed that 33% of the incidental findings needed further diagnostic investigation and possibly treatment by other specialities.

**Conclusion:**

Diagnostic assessment using orthodontic diagnostic X‑rays results in a high prevalence of incidental findings away from the dentition. Particularly in adults, a large number of incidental findings outside the dental/alveolar region may be expected on orthodontic diagnostic X‑rays. Thus, a structured approach during diagnostic assessment is required to minimise the extent to which incidental findings of clinical relevance are overlooked.

## Abstract (de)

**Ziel:**

Da sich immer mehr erwachsene Patienten in kieferorthopädischen Praxen vorstellen, ist mit einer Zunahme von Nebenbefunden auf diagnostischen Röntgenaufnahmen zu rechnen, die den Eckpfeiler der kieferorthopädischen Diagnostik darstellen. Daraus ergibt sich die klinisch relevante Frage, ob ein Alterseffekt bezüglich Prävalenz, Lokalisation und Schweregrad von Nebenbefunden in der kieferorthopädischen Röntgendiagnostik existiert.

**Material und Methoden:**

Die klinische, primär retrospektive Studie untersuchte pathologische Nebenbefunde von insgesamt 600 OPG(Orthopantomogramm)- und FRS(Fernröntgenseiten)-Aufnahmen anhand zweier Gruppen kieferorthopädischer Patienten (Gruppe I – 150 Kinder/Jugendliche; Alter 11,89 ± 2,47 Jahre; Gruppe II – 150 Erwachsene; 27,03 ± 10,42 Jahre). Prävalenz, Lokalisation und Schweregrad der Befunde wurden anhand eines Klassifikationsbogens aufgezeichnet. Die Befundung sah ein systematisches Vorgehen entlang von 9 Lokalisationen – Mandibula, Maxilla, Dentition, Nasennebenhöhlen, Kiefergelenk, Schädelbasis, Orbita, Halswirbelsäule, Weichgewebe – durch 3 erfahrene Prüfärzte vor.

**Ergebnisse:**

Insgesamt konnten 1458 Nebenbefunde bei allen Patienten nachgewiesen werden, wobei 66 % der Nebenbefunde abseits der Dentition auftraten. Es zeigte sich ein signifikanter Alterseffekt (*p* < 0,001) bezüglich der Prävalenz der Nebenbefunde (Gruppe II – Erwachsene 1026 Befunde – OPG: 566/FRS 460 vs. Gruppe I – Kinder/Jugendliche 432 Befunde – OPG: 221/FRS 211). Bezüglich der Lokalisation waren die Nebenbefunde bei Erwachsenen am häufigsten in den Regionen Dentition, Nasennebenhöhlen und Mandibula zu finden. Zudem ergaben sich in der Analyse der FRS-Aufnahmen bei Erwachsenen im Bereich der Schädelbasis und der Halswirbelsäule signifikant mehr Nebenbefunde (*p* < 0,001, *p* = 0,003). Die Kategorisierung nach Schweregrad der Nebenbefunde zeigte, dass 33 % der Nebenbefunde eine weiterführende Diagnostik und ggf. Therapie durch weitere Fachdisziplinen erforderlich machten.

**Zusammenfassung:**

Die kieferorthopädische Röntgendiagnostik bietet ein großes Befundungsareal, in dem Nebenbefunde abseits der Dentition mit hoher Prävalenz auftreten können. Insbesondere bei Erwachsenen ist von einer Vielzahl von Nebenbefunden auch außerhalb der dentalen/alveolären Region auf kieferorthopädischen Röntgenbildern auszugehen. Daher ist ein strukturiertes Vorgehen während der Befundung notwendig, um das Übersehen von Nebenbefunden mit klinischer Relevanz zu minimieren.

## Introduction

Orthodontic diagnostic X‑rays usually comprise an orthopantomogram (OPT) and a lateral cephalogram (LC) for assessing the principal orthodontic findings. In addition to the focal, principal findings relating to a diagnosis that clinically justified the radiation exposure, other findings can also be detected on X‑rays [1–7]. These are classified as incidental findings and are not directly related to, or the focus of, the primary diagnostic question. Incidental findings may be clinically nonrelevant or pathological [8–10]. Nonrelevant incidental findings have no implications for the patient and require no further diagnostic investigation. Pathological findings can, on the one hand, result in further diagnostic examination and, on the other hand, always have an influence on the subsequent treatment procedure. In orthodontics, too, a pathological incidental finding will have repercussions that may affect treatment decisions [11–14].

The prevalence of incidental findings varies on OPT and LC [5, 15, 16] and can range from purely dental anomalies through to pathomorphologies in distant regions of the jaw. There are reports in the literature about carious lesions and pulp changes, peridental changes to the alveolar process (apical radiolucencies/periodontal infractions) through to structural changes of the temporomandibular joint (TMJ), the maxillary sinuses, the cervical spine or cranial base, among others [5, 15, 17–22]. Rare brain tumours have also been described as shadowing on orthodontic LC in dramatic individual cases [23–26]. In such cases, early first assessment can markedly influence the prognosis of the treatment outcome for such neoplasms [14]. The importance of a thorough assessment of all X‑rays in the orthodontic context thus becomes obvious.

Incidental findings on dental radiographs have been published for orthodontic patients of different ages [2–5]. In the general dentistry setting, more incidental findings in the dental and extradental region have already been observed on radiological images in older people [27, 28]. Overall, the incidence of incidental findings appeared to increase with age.

However, a systematic work-up of incidental findings on orthodontic diagnostic X‑rays (OPT/LC) depending on age has not yet been described. This is gaining significance for practitioners, especially in view of the increased frequency of orthodontics in adults. For example, incidental findings can be detected on an OPT that have the highest clinical relevance, especially in older patients, such as opacities in the carotid area with a suspected arteriosclerotic cause [27, 29, 30].

Against this clinical background, the objectives of this study were

- Systematic analysis of incidental findings on orthodontic OPT and LC images,
- Evaluation of the influence of age on the prevalence of incidental findings,
- Assessment of the prevalence of incidental findings by region (maxilla, mandible, dentition, paranasal sinuses, temporomandibular joint, cranial base, orbit, cervical spine, soft tissues), and
- Classification of incidental findings according to clinical relevance.

## Patients and methods

This cross-sectional study was based on a retrospective approach. Approval was given by the institute’s own ethics committee (ethics number 1/11/16). The study was conducted according to the stipulations of the Declaration of Helsinki. The recommendations of the STROBE (Strengthening The Reporting of OBservational Studies in Epidemiology) statement were followed.

### Patients

A total of 300 study participants were randomly included from the patient base of the Department of Orthodontics, University Medical Centre Göttingen (UMG). The inclusion criteria were the following: patients aged > 6 years with two high-quality X‑rays (OPT and LC: Orthophos XG Plus DS/Ceph 9200 from Sirona Dental Systems GmbH, Bensheim, Germany) taken in the course of orthodontic diagnostic evaluation. All patients (in the case of minors, their parents or guardians) gave written consent to the general and data protection study conditions. The X‑rays had been taken according to the ALARA (as low as reasonably achievable) principle with an indication justifying radiation exposure.

The following criteria were set to exclude patients from the study: patients under the age of 6 years, patients with cleft lip and palate or with complex craniofacial syndromes and patients who did not sign a declaration of consent to the entire study protocol. The gender of the subjects played no role in the planned study.

The patients were classified into two age groups:

- Group I—150 children and adolescents, aged 6–17 years (mean 11.89 ± 2.47 years)
- Group II—150 adults, aged 18–72 years (mean 27.03 ±10.42 years)

### Methods

All X‑rays had been taken in the Radiology Department of the Centre for Dental, Oral and Maxillofacial Medicine, Medical Centre Göttingen, by expert radiography staff using the device Orthophos XG Plus DS/Ceph 9200 from Sirona Dental Systems GmbH (Bensheim, Germany) in the period from 09/2009 to 06/2017. OPTs of children and adolescents had the same size as OPTs of adults, no enlarged X‑rays were included. All LCs had a size of 18 × 24 cm and showed the anterior head. Three examiners (DK, PS, CV) assessed all 600 images together in the period from 07/2017 to 05/2018. Unclear findings were further clarified by an experienced radiologist (JSS). All the X‑rays were analysed on the RadiForce MX215 monitor (Eizo®, Hokusan, Japan) licensed in accordance with DIN V 6868-157 for dental assessment within the quality assurance line, using the imaging software Sidexis XG 2.65 (Sirona Dental Systems GmbH, Bensheim, Germany). Calibration of the examiners took place in a pilot phase, where each examiner analysed 30 X-rays which had previously been assessed by an experienced radiologist (JSS).

**Fig. 1:**
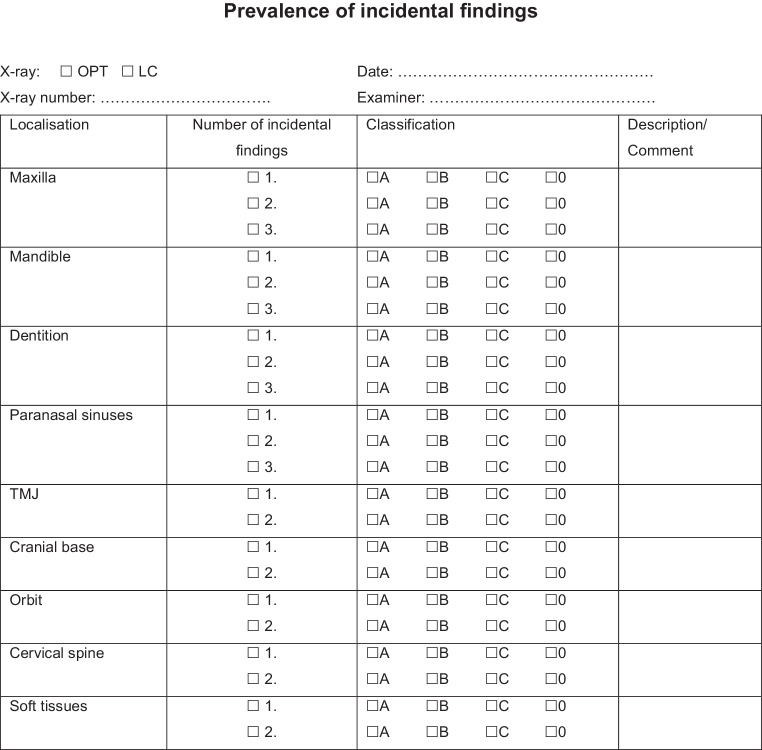
Chart of the classification system for assessment of all X‑rays. *TMJ* temporomandibular joint, *LC* lateral cephalogram, *OPT* orthopantomogram Darstellung des Klassifikationsschemas zur Befundung aller Röntgenbilder. *TMJ* Kiefergelenk, *LC* Fernröntgenseitenbild, *OPT *Orthopantomogramm

All X‑rays were analysed according to a structured protocol involving documentation of (I.) localisation, (II.) prevalence and (III.) classification of each finding (Fig. 1):

I. Localisation: Each finding was assigned to one of nine localisations: maxilla, mandible, dentition, paranasal sinuses, TMJ, cranial base, orbit, cervical spine and soft tissues;
II. Prevalence: Number of findings per localisation was totalled—same kind of pathologies (e.g. generalised root resorptions) were combined into one incidental finding and rated once. Missing teeth, of both genetic and acquired origin, were combined under the heading “reduced number of teeth”;
III. Classification: The classification scheme was based on the BI-RADS® scoring system, which has been used in breast cancer diagnosis for years: using a four-tiered model, each finding was rated from 0: no classification possible through to C: potentially malignant (Table 1).

**Table 1:** Classification based on the BI-RADS® scoring system An das BI-RADS®-Scoringsystem angelehnte Klassifikation

Group	Score
0	No score possible (= at the current time, no score/classification possible. For example, due to technical errors, artefacts)
A	Definitely benign: needs no further diagnostic investigation after the present imaging; the diagnosis is clear after the first scan
B	Primarily unclear, intermediate: no clear finding is established after the present imaging. Needs further diagnostic investigation—later likely to prove benign
C	Clearly treatment-changing or potentially malignant: after the present imaging, needs additional diagnostic investigation, possibly also biopsy investigation of the incidental finding. A change of treatment and of patient management is probable

### Statistical analysis

The statistical analysis provided for descriptive, exploratory and inductive analysis of the data. The databases created in Excel (Excel Version 16.19, 2018; Microsoft Corp., Redmond, WA, USA) were merged and analysed using the statistical programming language R 3.5.0 in R‑Studio®, 2016 (R-Studio Inc., Boston, MA, USA). In terms of descriptive statistics, mean, standard deviation, minimum and maximum of the available dataset were determined in the various analysis categories (age group, localisation and classification).

The independent variables were tested for a sufficient Gaussian/normal distribution using the Kolmogorov–Smirnov goodness-of-fit test. Given a normal distribution, the statistical significance was analysed by the t‑test for unpaired groups between the age groups children/adolescents vs. adults in the OPT and the LC. The level of significance with respect to the character of the study was set at *p* < 0.05. Corrections for multiple tests were not carried out in view of the exploratory character of the study and the use of a model including all relevant variables.

## Results

**Fig. 2:**
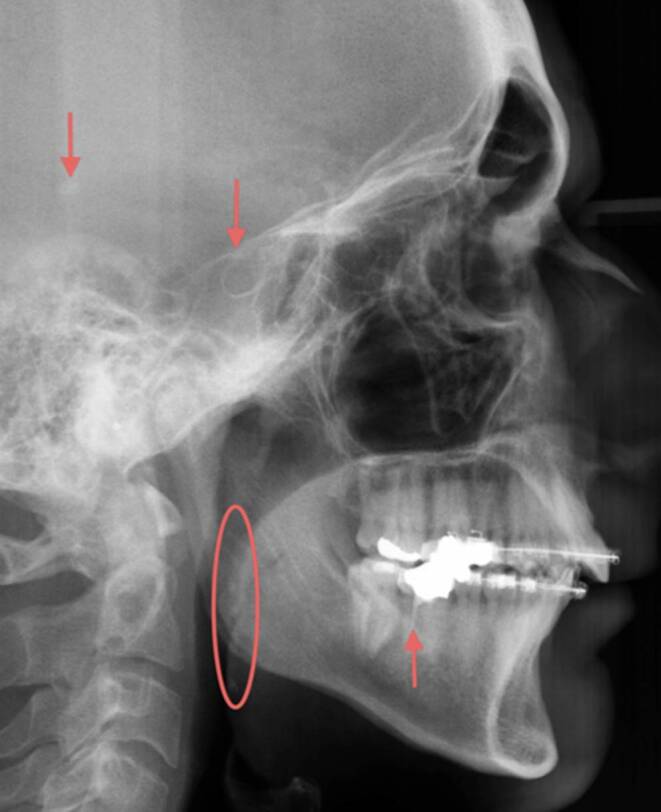
Lateral cephalogram (LC) of a 47-year-old man with various incidental findings (*arrows*: sella turcica bridging, insufficient root filling, intracranial calcification and *circled*: suspected tonsil stones) FRS (Fernröntgenseitenbild) eines 47-jährigen Patienten mit diversen Nebenbefunden (*Pfeile*: Sella-turcica-Brücke, insuffiziente Wurzelfüllung, intrakranielle Kalzifizierung und *eingekreist*: Verdacht auf Tonsillensteine)

**Fig. 3:**
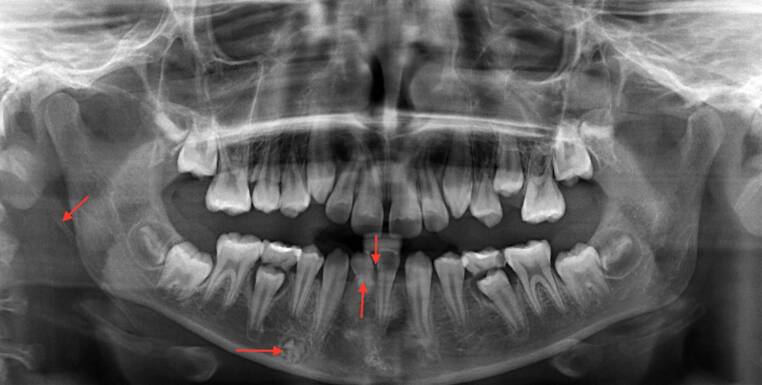
Orthopantomogram (OPT) of an 11-year-old boy with various incidental findings (*arrows*: reduced tooth number, dilaceration at tooth 42, suspected odontomas region 44 and calcified right-sided stylohyoid ligament) Orthopantomogramm (OPG) eines 11-jährigen Patienten mit diversen Nebenbefunden (*Pfeile*: Zahnunterzahl, Dilazeration am Zahn 42, Verdacht auf Odontome Regio 44 und kalzifiziertes Ligamentum stylohoideum rechts)

In the course of the study, a total of 1458 incidental findings (423 incidental findings in children and adolescents and 1026 in adults) were detected on the orthodontic diagnostic X‑rays (OPT and LC) in 289 of the 300 study participants (Table 2). This gives an overall prevalence of 4.86 incidental findings per patient. As an example, Figs. 2 and 3 show two X‑rays from the present study with the incidental findings marked.

**Table 2:** Descriptive statistics and t‑test regarding the prevalence of all incidental findings depending on age group (children/adolescents vs. adults); significance for *p* < 0.05 Deskriptive Statistik und t‑test bezüglich der Prävalenz aller Nebenbefunde in Abhängigkeit der Altersgruppe (Kinder/Jugendliche vs. Erwachsene); Signifikanz für *p* < 0,05

Diagnostic X‑rays	Group I (*N* = 150) (children/adolescents)					Group II (*N* = 150) (adults)					Unpaired t‑test
	Number of incidental findings										
	Overall	Mean (SD)	Min	Max	95% CI	Overall	Mean (SD)	Min	Max	95%CI	*p*
OPT	221	1.48 ±1.3	0	7	1.27–1.69	566	3.74 ±2.3	0	13	3.37–4.1	< 0.001 ***
LC	211	1.41 ±1.1	0	5	1.23–1.59	460	3.07 ± 2	0	11	2.75–3.39	< 0.001 ***
All	432	–	–	–	–	1026	–	–	–	–	–

*n.* *s. *not significant, *95% CI* 95% confidence interval, *Min* minimum, *Max* maximum, *SD* standard deviation, *OPT* orthopantomogram, *LC* lateral cephalogram

* *p* < 0.05, ** *p* < 0.01, *** *p* < 0.001

The prevalence of incidental findings was significantly higher in adults than in children and adolescents (*p* < 0.001). On the OPT, adults demonstrated a mean of 3.74 (±2.3) findings compared to 1.48 (±1.3) in children and adolescents. On LC, adult patients had a mean of 3.07 (±2) findings versus 1.41 (±1.1) in children (Table 2). Among the children and adolescents, 10 patients had no incidental findings on the OPT or the LC. Only 1 patient in the adult group had no incidental finding detectable on the X‑rays.

The frequency of the incidental findings on both, the OPT and the LC, being localized extradental or dental varied with regard to the overall number of incidental findings: the ratio of the incidence of findings was increased in extradental regions for both children/adolescents (extradental findings—OPT: 52.9%; LC: 76.8%) and adults (extradental findings—OPT: 60.1%; LC: 74.4%) (Tables 3 and 4).

**Table 3:** Descriptive statistics and t‑test regarding the localisation of all incidental findings (dental vs. extradental findings and subclassification) depending on age group (children/adolescents vs. adults) in OPT Deskriptive Statistik und t‑test bezüglich der Lokalisation aller Nebenbefunde (dentale vs. extradentale Befunde und Subklassifikation) in Abhängigkeit der Altersgruppe (Kinder/Jugendliche vs. Erwachsene) im OPG

Diagnostic X‑rays	Localisation	Group I children/adolescents (*N* = 150)			Group II adults (*N* = 150)			Unpaired t‑test
		Number of incidental findings						
		*n*	(∅)	%	*n*	(∅)	%	*p*
OPT	Dental findings	104	(0.7)	47.1	226	(1.47)	39.9	< 0.001 ***
	Extradental findings	117	(0.78)	52.9	340	(2.26)	60.1	< 0.001 ***
	*Subclassification extradental findings*							–
	Mandible	19	(0.13)	8.6	103	(0.7)	18.2	< 0.001 ***
	Maxilla	9	(0.06)	4.1	69	(0.46)	12.2	< 0.001 ***
	Paranasal sinuses	45	(0.3)	20.4	92	(0.61)	16.3	< 0.001 ***
	TMJ	17	(0.11)	7.7	35	(0.23)	6.2	0.006 **
	Cranial base	3	(0.02)	1.4	0	(0)	0	> 0.05 n. s.
	Orbit	0	(0)	0	2	(0.01)	0.4	> 0.05 n. s.
	Cervical spine	0	(0)	0	0	(0)	0	–
	Soft tissues	24	(0.16)	10.9	39	(0.25)	6.9	> 0.05 n. s.

*n.* *s.* not significant, *TMJ* temporomandibular joint, *(∅)* findings per patient in age group; % calculated proportion of total findings for age group, *OPT* orthopantomogram

* *p* < 0.05, ** *p* < 0.01, *** *p* < 0.001

**Table 4:** Descriptive statistics and t‑test regarding the localisation of all incidental findings (dental vs. extradental findings and subclassification) depending on age group (children/adolescents vs. adults) in LC Deskriptive Statistik und t‑test bezüglich der Lokalisation aller Nebenbefunde (dentale vs. extradentale Befunde und Subklassifikation) in Abhängigkeit der Altersgruppe (Kinder/Jugendliche vs. Erwachsene) im FRS

Diagnostic X‑rays	Localisation	Group I children/adolescents (*N* = 150)			Group II adults (*N* = 150)			Unpaired t‑test
		Number of incidental findings						
		*n*	(∅)	%	*n*	(∅)	%	*p*
LC	Dental findings	49	(0.33)	23.22	118	(0.79)	25.65	< 0.001 ***
	Extradental findings	162	(1.08)	76.78	342	(2.28)	74.35	< 0.001 ***
	*Subclassification extradental findings*							–
	Mandible	7	(0.05)	3.3	45	(0.3)	9.8	< 0.001 ***
	Maxilla	5	(0.03)	2.4	41	(0.27)	8.9	< 0.001 ***
	Paranasal sinuses	36	(0.24)	17.1	93	(0.62)	20.2	< 0.001 ***
	TMJ	0	(0)	0	6	(0.04)	1.3	0.014 *
	Cranial base	60	(0.4)	28.4	97	(0.65)	21.1	< 0.001 ***
	Orbit	2	(0.01)	0.9	0	(0)	0	> 0.05 n. s.
	Cervical spine	13	(0.09)	6.2	32	(0.21)	7.0	0.003 **
	Soft tissues	39	(0.26)	18.5	28	(0.19)	6.1	> 0.05 n. s.

*n.* *s.* not significant, *TMJ* temporomandibular joint, *(∅)* findings per patient in age group,% calculated proportion of total findings for age group, *LC* lateral cephalogram

* *p* < 0.05, ** *p* < 0.01, *** *p* < 0.001

Analysis of the subclassification of the extradental findings on the OPT between the age groups showed that most of the incidental findings were located in the paranasal sinuses in adolescents at 20.4%, whereas in adults the highest number of findings was in the mandible at 18.2%. However, incidental findings were also localised in regions topographically distant from the teeth and jaw, such as in the “soft tissues” region, accounting for as much as 10.9% (children/adolescents) and 6.9% (adults).

Significantly more findings on the OPT occurred in both the mandible (*p* < 0.001) and the maxilla (*p* < 0.001) in the adult group, whereas in the children/adolescent group there were more incidental findings in the region of the paranasal sinuses (*p* < 0.001) and TMJ (*p* < 0.006; Table 3).

Analysis of the LC images in turn revealed that, in both age groups, the highest number of extradental incidental findings was recorded far away from the area of the jaws in the cranial base: 28.4% in children/adolescents and 21.1% in adults. Furthermore, comparison of the extradental findings on the LC between the two age groups showed that adults had significantly more incidental findings in all regions (mandible: *p* < 0.001; maxilla: *p* < 0.001; paranasal sinuses: *p* < 0.001; TMJ: *p* *=* 0.014; cranial base: *p* *<* 0.001; cervical spine: *p* *=* 0.003) except for the orbit and the soft tissues (Table 4). Table 5 gives a complete detailed overview of all the incidental findings depending on localisation.

**Table 5:** Detailed overview of incidental findings from both forms of imaging (OPT/LC combined) in the nine localisations Detaillierte Übersicht der Nebenbefunde aus beiden Bildgebungen (OPG/FRS summiert) in den 9 Lokalisationen

Incidental findings in anatomical categories	*n*	(%)
**Dental findings**	497	(34.1)
Caries	130	(8.9)
Reduced tooth number	115	(7.9)
Impacted tooth	108	(7.4)
Foreign body, unclear classification	69	(4.7)
Root resorption	34	(2.3)
Calculus	7	(0.5)
Primary tooth root persistence	3	(0.2)
Insufficient root filling	2	(0.1)
Other	29	(2)
**Extradental findings**	961	(65.9)
**Maxilla**	124	(8.5)
Marginal periodontitis	56	(3.8)
Apical radiolucency	33	(2.3)
Other radiolucency	12	(0.8)
Foreign body, uncertain classification	11	(0.8)
Follicular cyst	3	(0.2)
Bone atrophy	4	(0.3)
Other	5	(0.3)
**Mandible**	174	(11.9)
Marginal periodontitis	41	(2.8)
Apical radiolucency	31	(2.1)
Other unclear opacity	29	(2)
Other unclear radiolucency	24	(1.6)
Foreign body	20	(1.4)
Primary tooth persistence	6	(0.4)
Mental foramen	7	(0.5)
Other	16	(1.1)
**Paranasal sinuses**	266	(18.2)
Deep recess	125	(8.6)
Opacity	84	(5.8)
Pronounced septation	16	(1.1)
Frontal sinus hypoplasia	20	(1.4)
Other	21	(1.4)
**TMJ**	58	(4)
Asymmetry	32	(2.1)
Pronounced anomaly of morphology	13	(0.9)
Other	13	(0.9)
**Cranial base**	160	(11)
Tendency to sella turcica bridging	70	(4.8)
Sella turcica bridging	42	(2.9)
Macro-sella	17	(1.1)
Opacity unclear	15	(1)
Other	16	(1.1)
**Orbit**	4	(0.3)
Opacity of unclear origin	2	(0.1)
Asymmetry	2	(0.1)
**Cervical spine**	45	(3.1)
Ponticulus posticus	17	(1.2)
Fusion	15	(1)
Shape anomaly	8	(0.6)
Other	5	(0.3)
**Soft tissues**	130	(9)
Tonsillar hyperplasia	56	(3.8)
Stylohyoid ligament (calcified)	29	(2)
Foreign body, uncertain classification	18	(1.2)
Carotid artery (calcified)	6	(0.4)
Tonsil stones	6	(0.4)
Other	15	(1)
**Total**	1458	(100)

*TMJ* temporomandibular joint, *n *number of incidental findings, *(%) *percentage of 1458 incidental findings, *OPT* orthopantomogram, *LC* lateral cephalogram

Analysis of the classification of all the incidental findings depending on age group illustrates the frequencies of A and B findings on the OPT and the LC, as shown in Tables 6, 7, 8 and 9. Overall, no findings were assigned to classifications C or 0 for either the children/adolescents or the adults. Comparison of the groups revealed a significant difference for the OPT, with a significantly higher number of A findings for children/adolescents and significantly more B findings in the adult group. The LC images showed a significantly slightly higher number of A findings in adults (Tables 6 and 8, part I). Most of the B findings in the children/adolescent group were assigned to the dentition in both X‑rays, the LC and the OPT. In adults, however, B findings were found primarily in the maxilla and the mandible region (Tables 7 and 9, part II).

**Table 6:** Descriptive statistics and t‑test regarding the classification of all incidental findings (A/B findings) depending on age group (children/adolescents vs. adults) in OPT; overall, no findings were assigned to 0 classification. For this reason, the 0 classifications are not listed in the following presentation of results Deskriptive Statistik und t‑test bezüglich der Klassifikation aller Nebenbefunde (A-/B-Befunde) in Abhängigkeit der Altersgruppe (Kinder/Jugendliche vs. Erwachsene) im OPG; insgesamt wurde kein Befund der Klassifikation 0 zugeordnet. Aus diesem Grund werden in der folgenden Darstellung der Ergebnisse die Klassifikationen 0 nicht aufgeführt

Diagnostic X‑rays	Classification	Group I children/adolescents (*N* = 150)			Group II adults (*N* = 150)			Unpaired t‑test
		Number of incidental findings						
		*n*	(∅)	%	*n*	(∅)	%	*p*
OPT	A findings	135	(0.91)	61.08	323	(2.14)	57.07	<0.001 ***
	B findings	86	(0.28)	38.91	243	(0.38)	42.93	0.007 **

*(∅)* finding per patient in age group, *%* calculated proportion of total findings, *OPT* orthopantomogram

* *p* < 0.05, ** *p* < 0.01, *** *p* < 0.001

**Table 7:** Description regarding the classification of all incidental findings (A/B findings) according to localisation for OPT Übersicht der Klassifikation der Nebenbefunde (A-/B-Befunde) nach ihrer Lokalisation im OPG

Localisation	Classification	Group I children/adolescents (*N* = 150)	Group II adults (*N* = 150)
		Number of incidental findings	
		*n*	*n*
Dental	A	63	173
	B	41	64
Mandible	A	13	33
	B	6	70
Maxilla	A	2	12
	B	7	57
Paranasal sinuses	A	17	56
	B	28	36
TMJ	A	15	31
	B	2	4
Cranial base	A	3	0
	B	0	0
Orbit	A	0	1
	B	0	1
Cervical spine	A	0	0
	B	0	0
Soft tissues	A	22	28
	B	2	11

*TMJ* temporomandibular joint, *OPT* orthopantomogram

**Table 8:** Descriptive statistics and t‑test regarding the classification of all incidental findings (A/B findings) depending on age group (children/adolescents vs. adults) in LC; overall, no findings were assigned to 0 classification. For this reason, the 0 classifications are not listed in the following presentation of results Deskriptive Statistik und t‑test bezüglich der Klassifikation aller Nebenbefunde (A-/B-Befunde) in Abhängigkeit der Altersgruppe (Kinder/Jugendliche vs. Erwachsene) im FRS; insgesamt wurde kein Befund der Klassifikation 0 zugeordnet. Aus diesem Grund werden in der folgenden Darstellung der Ergebnisse die Klassifikationen 0 nicht aufgeführt

Diagnostic X‑rays	Classification	Group I children/adolescents (*N* = 150)			Group II adults (*N* = 150)			Unpaired t‑test
		Number of incidental findings						
		*n*	(∅)	%	*n*	(∅)	%	*p*
LC	A findings	161	(1.07)	76.3	355	(2.36)	77.17	<0.001 ***
	B findings	50	(0.16)	23.7	105	(0.14)	22.83	>0.05 n. s.

*(∅)* finding per patient in age group, *%* calculated proportion of total findings, *LC* lateral cephalogram

* *p* < 0.05, ** *p* < 0.01, *** *p* < 0.001

**Table 9:** Description regarding the classification of all incidental findings (A/B findings) according to localisation for LC Übersicht der Klassifikation der Nebenbefunde (A-/B-Befunde) nach ihrer Lokalisation im FRS

Localisation	Classification	Group I children/adolescents (*N* = 150)	Group II adults (*N* = 150)
		Number of incidental findings	
		*n*	*n*
Dental	A	26	105
	B	23	13
Mandible	A	4	22
	B	3	24
Maxilla	A	0	8
	B	5	32
Paranasal sinuses	A	28	78
	B	8	15
TMJ	A	0	2
	B	0	4
Cranial base	A	56	86
	B	5	11
Orbit	A	2	0
	B	0	0
Cervical spine	A	12	31
	B	1	1
Soft tissues	A	33	23
	B	5	5

*TMJ* temporomandibular joint, *LC* lateral cephalogram

## Discussion

Only a few clinically representative analyses regarding potential incidental findings within radiological diagnostics have hitherto been published in the orthodontic literature [2, 31–33]. Isolated publications give an overview of the general prevalence of incidental findings in the different anatomical areas of the maxillofacial/head region [2, 4, 5]. For example, Hernández et al. [5] gave a descriptive report of the age effect on the number of incidental findings in the patients they studied. None of the quoted studies encompassed all relevant anatomical regions or a classification of the incidental findings.

The age range of the patients analysed in the present study can be regarded as representative of the average orthodontic practice. The group breakdown revealed two age peaks, corresponding to the typical start time for orthodontic treatments in children and adults respectively. Orthodontic treatment of children and adolescents usually starts in the second phase of the mixed dentition and this explains the first age peak at 10–13 years [34, 35]. A second peak was in the third decade of life and corresponds to the average time period for the initiation of adult therapy [36]. Against the background of a steady growth in the number of adults attending orthodontic practices [37], the significantly increased prevalence of incidental findings in adult patients shows the need for a systematic analysis of orthodontic diagnostic X‑rays depending on patient age. The examiner selection, who assessed the X‑rays together, was similar to the study design by Bondemark et al. [4]. However, the 496 images investigated by Bodemark et al. were examined by only two examiners and noticeable results were assessed by an experienced third examiner. Moreover, the findings of Hernández et al. [5] were subjectified only by one clinician. These authors examined significantly more OPG and LC images than in the present study, but the risk of overlooking findings was probably increased due the fact that the X‑rays were examined by only one investigator.

The subdivision into dental findings versus extradental findings showed a higher prevalence of incidental findings in the extradental category for both age groups (Tables 3 and 4). This difference was very apparent in the LC analysis, with more than three times the number of extradental incidental findings. To date, there are only a few clinically representative analyses of incidental findings distant from the dental focus, away from the typical regions of interest [2, 31–33]. Furthermore, no data have been reported regarding a uniform analysis of the general prevalence of incidental findings in different regions [2, 4, 5]. In addition, the quoted studies differ in terms of subdivision of the localisations and the age range of the patients studied, making data comparison difficult.

The results of this study showed that adults were presented with more incidental findings than children and adolescents by a factor of 2.5 (OPT) and 2.2 (LC) respectively. Other studies have also demonstrated an age effect [5]. Compared to previous studies [2, 4, 5], a higher prevalence of incidental findings could be reported in the present study. For instance, a cross-sectional study of 782 patients with 1887 X‑rays showed markedly fewer incidental findings with 0.88 findings per study participant [5]. One explanation might lie in the diagnostic regime of the present study: unlike the previously published results, in this study all 600 X‑rays were analysed by three examiners using a predefined assessment form after a calibration phase. Generally speaking, the numbers from the previously published data on incidental findings in orthodontic diagnostic X‑rays being performed without structured assessment might have underestimated the true diagnoses. In the present study, markedly more findings—especially in the adult group—were described for both the OPT and the LC. This is a further endorsement of two basic principles to be heeded by practising orthodontists. Firstly, it shows the need for ubiquitous analysis of all regions depicted on the particular images. Secondly, more incidental findings located outside the dental region are to be expected with increasing patient age.

Furthermore, the detailed subclassification of the extradental region in the LC showed that adult patients had significantly more findings than children/adolescents in almost all regions (except orbit, soft tissues and cranial base). The largest proportion of incidental findings were found in the cranial base region (children/adolescents: 28.4%, adults: 21.1%)—the majority being caused by changes to the sella turcica (tendency to sella turcica bridging, sella turcica bridging, macro-sella, etc.). It was also interesting that 6.2% (children/adolescents) and 7% (adults) of incidental findings were localised in the region of the cervical spine; among these, ponticulus posticus was most commonly diagnosed. This pathomorphology is a finding which does not lie within the therapeutic scope of orthodontists, but is nevertheless depicted in orthodontic diagnostic imaging. Even though ponticulus posticus has generally been described as an anatomical variant, associations with clinical conditions such as chronic tension headache, migraine without aura, cervical pain syndrome through to sudden hearing loss are nonetheless reported in current oral medicine literature [38]. As a result, such variants should also be identified during the course of a thorough X‑ray assessment.

In the subclassification of the extradental regions on the OPT, the significantly higher number of incidental findings in the paranasal sinuses and TMJ regions in adult patients is particularly noticeable. In a previous study, a 41- to 60-year-old age group was particularly striking with a very high prevalence of paranasal sinus pathologies [39]. Gracco et al. [39] found similar age differences in the area of the paranasal sinuses in cone beam computed tomographic (CBCT) images as those in the present study. Edwards et al. [7] on CBCT images and Hernández et al. [5] also observed numerous physiological remodelling (flat condylar edges, subchondral sclerosis) and degenerative changes (osteophytes, erosions) affecting the temporomandibular joint (TMJ). Accordingly, in the areas of the paranasal sinuses and the TMJ, a markedly higher prevalence of pathological anomalies can therefore be assumed in adults. Although two-dimensional images reveal many findings, CBCT is in many ways superior to two-dimensional images [40, 41]. Whether the indication for a CBCT diagnosis is made, depends on the expertise of the examiner [42]. Compared to the two-dimensional OPT recording, the CBCT data record is convincing with its true-to-geometry and distortion-free reproduction [43]. With regard to the present study, it is questionable whether the findings could be assigned to the correct locations due to overlapping. Nevertheless, it is important to document the findings as standardised as possible and, especially if the findings or the localisation are ambiguous, to carry out further diagnostics, which is also underlined by the results of our investigation.

The record form provided the examiners with a structured sequence for assessing findings. This means, firstly, that the possibility of overlooking findings is greatly reduced [44, 45] and secondly, that a standardisation of the results is achieved. Structured and standardised diagnosis and documentation of X‑ray diagnosis is meanwhile applied in all medical specialities [46].

It becomes clear that such a systematic procedure for every X‑ray assessment would be advantageous for orthodontists in clinical practice. Furthermore, transferring the BI-RADS® classification to the head and neck region makes it easier to classify pathological anomalies. The BI-RADS® scoring system is an internationally recognized instrument in breast cancer diagnosis [47, 48]. The latest version of the BI-RADS® (2013) [49] comprises 6 categories, with levels 3–6 dealing with the percentage probability of malignancy. Since malignant findings are extremely rare in orthodontic and dental practice [50, 51], BI-RADS® (2013) [49] was rated as inappropriate for the present study. The categorisation of the present study is based on the BI-RADS® version from 1998, which summarizes malignant findings in just one category [52].

Use of this instrument made it possible to classify the results of this study into two categories (category A and category B). Routine classification of findings improves early detection of findings requiring further investigation [51, 53]. In many cases, early detection can improve the patient’s prognosis. Progression of arteriosclerotic pathologies for example can be slowed down with medication [53, 54]: beginning treatment early can lead to a better prognosis and successful course of therapy [53]. Bengtsson et al. [29] and Uthman and Al-Saffar [27] found a significant association between carotid calcifications in the OPG and the occurrence of a stroke or ischemic heart disease in patients older than 40 years. In the present study, category B findings were classified as requiring investigation. No clear finding was determined, and further diagnostic investigation was necessary. The high prevalence of B‑classified findings in both forms of imaging in this study further reinforces the importance of a standardised assessment and of a pathological scoring of every finding.

## Conclusion

A structured diagnostic procedure of orthodontic X‑rays showed a high prevalence of incidental findings, also outside the primary dental focus. Adults, in particular, demonstrated markedly more incidental findings per image in the extradental regions than children/adolescents. The need for a thorough and optimal assessment of OPT and LC images for the early diagnosis of incidental findings by practising orthodontists becomes clear. The results could be incorporated into a preliminary draft directive for studying radiographic images which are produced in the course of orthodontic treatment planning regarding incidental findings. However, further studies should be carried out in order to examine the concrete procedure of standardisation more closely.

## References

[1] Pakbaznejad Esmaeili E, Ekholm M, Haukka J (2016). Type and location of findings in dental panoramic tomographs in 7–12-year-old orthodontic patients. Acta Odontol Scand.

[2] Tetradis S, Kantor ML (1999). Prevalence of skeletal and dental anomalies and normal variants seen in cephalometric and other radiographs of orthodontic patients. Am J Orthod Dentofacial Orthop.

[3] Kuhlberg AJ, Norton LA (2003). Pathologic findings in orthodontic radiographic images. Am J Orthod Dentofacial Orthop.

[4] Bondemark L, Jeppsson M, Lindh-Ingildsen L (2006). Angle Orthod.

[5] Hernández G, Plaza SP, Cifuentes D (2018). Incidental findings in pre-orthodontic treatment radiographs. Int Dent J.

[6] van Waes HJM, Stöckli PW (2001). Periorale und orale Symptome der Kinderkrankheiten. Kinderzahnmedizin. Farbatlanten der Zahnmedizin.

[7] Edwards R, Alsufyani N, Heo G (2014). The frequency and nature of incidental findings in large-field cone beam computed tomography scans of an orthodontic sample. Prog Orthod.

[8] Marx RE, Sawatari Y, Fortin M (2005). Bisphosphonate-induced exposed bone (osteonecrosis/osteopetrosis) of the jaws: risk factors, recognition, prevention, and treatment. J Oral Maxillofac Surg.

[9] Jacobsen C, Kruse Gujer A, Jacobsen C, Grätz KW (2013). Odontogene Tumoren. Facharztwissen Mund‑, Kiefer- und Gesichtschirurgie.

[10] Büttner M (2010). Gutartige Tumore des Gesichtsschädels. Schweiz Z Onkol.

[11] Thoma M (2010). Odontogene Infektionen.

[12] Kawakami M, Takano-Yamamoto T (1997). Orthodontic treatment of a patient with hypophosphatemic vitamin D-resistant rickets. ASDC J Dent Child.

[13] Linglart A, Biosse-Duplan M, Briot K (2014). Therapeutic management of hypophosphatemic rickets from infancy to adulthood. Endocr Connect.

[14] Alkofide E (2001). Pituitary adenoma: a cephalometric finding. Am J Orthod Dentofacial Orthop.

[15] Stošić E (2014). Zufallsbefunde im Orthopantomogramm: Eine Übersicht über die alters- und geschlechtsspezifische Verteilung.

[16] Teiser H (2009). Haupt- und Nebenbefunde bei der Auswertung von Panoramaschichtaufnahmen.

[17] Donald PM, Nagraj SK, Pallivathukkal RG (2017). Ponticulus posticus of atlas vertebrae: an incidental finding in Malaysian orthodontic patients. BMJ Case Rep.

[18] Meyer-Marcotty P, Reuther T, Stellzig-Eisenhauer A (2010). Bridging of the sella turcica in skeletal Class III subjects. Eur J Orthod.

[19] Viktorov Y (2006). Prävalenz von apikalen Parodontitiden sowie die Häufigkeit und Qualität endodontischer Behandlungen in einer Berliner Population.

[20] Pasler FA, Visser H (2003). Röntgendiagnostik bildgebender Parodontopathien und Entzündungen der Kiefer. Taschenatlas der zahnärztlichen Radiologie.

[21] Klenke D, Quast A, Prelog M (2018). TMJ pathomorphology in patients with JIA-radiographic parameters for early diagnosis. Head Face Med.

[22] Hülsmann M (1997). Dens invaginatus: aetiology, classification, prevalence, diagnosis, and treatment considerations. Int Endod J.

[23] Thurn P, Bücheler E, Lackner K-J (1998). Einführung in die radiologische Diagnostik.

[24] Oestmann J-W (2005). Radiologie. Vom Fall zur Diagnose.

[25] Rother UJ (2006). Veränderungen und Erkrankungen der Zahnhartgewebe, der Pulpa und des Parodontiums. Moderne bildgebende Diagnostik in der Zahn‑, Mund- und Kieferheilkunde.

[26] Loewenhardt B (2006). Bildgebende Diagnostik.

[27] Uthman AT, Al-Saffar AB (2008). Prevalence in digital panoramic radiographs of carotid area calcification among Iraqi individuals with stroke-related disease. Oral Surg Oral Med Oral Pathol Oral Radiol Endod.

[28] Ribeiro A, Keat R, Khalid S (2018). Prevalence of calcifications in soft tissues visible on a dental pantomogram: a retrospective analysis. J Stomatol Oral Maxillofac Surg.

[29] Bengtsson VW, Persson GR, Berglund J (2019). Carotid calcifications in panoramic radiographs are associated with future stroke or ischemic heart diseases: a long-term follow-up study. Clin Oral Investig.

[30] Nasseh I, Aoun G (2018). Carotid artery calcification: a digital panoramic-based study. Diseases.

[31] Schulz D, Bührmann K (1987). Pathologische Veränderungen der Kieferhöhle – wichtige Nebenbefunde bei der kieferorthopädischen Röntgendiagnostik (Pathological changes in the maxillary sinus—important secondary findings in orthodontic x-ray diagnosis). Fortschr Kieferorthop.

[32] Soni P, Sharma V, Sengupta J (2008). Cervical vertebrae anomalies-incidental findings on lateral cephalograms. Angle Orthod.

[33] Mudit G, Srinivas K, Satheesha R (2014). Retrospective analysis of ponticulus posticus in Indian orthodontic patients-a lateral cephalometric study. Ethiop J Health Sci.

[34] Kahl-Nieke B (2010). Einführung in die Kieferorthopädie. Diagnostik, Behandlungsplanung, Therapie.

[35] Harzer W (2021). Kieferorthopädie. Kieferorthopädische Betreuung durch den Hauszahnarzt.

[36] Evans S (2018) Orthodontic treatment increases popularity amongst adults. https://dentistry.co.uk/2018/05/09/orthodontic-treatment-increases-popularity-amongst-adults/. Accessed 3 Nov 2021

[37] Meyer-Marcotty P (2015). Kieferorthopädie als Segment eines interdisziplinären Behandlungskonzeptes bei ausgeprägter parodontaler Schädigung. Quintessenz.

[38] Tambawala SS, Karjodkar FR, Sansare K (2017). Prevalence of ponticulus posticus on lateral cephalometric radiographs, its association with cervicogenic headache and a review of literature. World Neurosurg.

[39] Gracco A, Incerti Parenti S, Ioele C (2012). Prevalence of incidental maxillary sinus findings in Italian orthodontic patients: a retrospective cone-beam computed tomography study. Korean J Orthod.

[40] Maddalone M, Bonfanti E, Pellegatta A (2019). Digital orthopantomography vs cone beam computed tomography-part 1: detection of periapical lesions. J Contemp Dent Pract.

[41] Ristow O, Schnug G, Smielowksi M (2021). Diagnostic accuracy comparing OPT and CBCT in the detection of non-vital bone changes before tooth extractions in patients with antiresorptive intake. Oral Dis.

[42] Radic J, Patcas R, Stadlinger B (2018). Do we need CBCTs for sufficient diagnostics?-dentist-related factors. Int J Implant Dent.

[43] Zöller JE, Neugebauer J (2013). Digitale Volumentomografie in der Zahn‑, Mund- und Kieferheilkunde. Grundlagen, Diagnostik und Behandlungsplanung.

[44] Classen M, Wagner F, Swobodnik W (1991). Electronic data base in gastroenterological endoscopy. Endoscopy.

[45] Dirsch I (1987). Befunddokumentation in der gynäkologischen Morphologie, Entwurf eines computergerechten Dokumentationssystems.

[46] ESR (2011) Good practice for radiological reporting. Guidelines from the European Society of Radiology (ESR). Insights Imaging 2(2): 93–96. 10.1007/s13244-011-0066-7 10.1007/s13244-011-0066-7 PMC3259387 22347937

[47] Balleyguier C, Ayadi S, van Nguyen K (2007). BIRADS classification in mammography. Eur J Radiol.

[48] Tardivon AA, Athanasiou A, Thibault F (2007). Breast imaging and reporting data system (BIRADS) magnetic resonance imaging illustrated cases. Eur J Radiol.

[49] D’Orsi CJ, Sickles EA, Mendelson EB, Morris EA (2013). Breast Imaging Reporting and Data System. Richtlinien zu Befundung, Handlungsempfehlungen und Monitoring = Richtlinien zu Befundung, Handlungsempfehlungen und Monitoring.

[50] Moffitt AH (2011). Discovery of pathologies by orthodontists on lateral cephalograms. Angle Orthod.

[51] Jundt G, Reichart PA (2008). Maligne odontogene Tumoren (Malignant odontogenic tumors). Pathologe.

[52] American College of Radiology (1998). Illustrated breast imaging reporting and data system (Illustrated BI-RADSTM).

[53] Windler E, Beil F‑U, Klose G (2018). Lipidsenkende Therapie im Alter: Wer profitiert von welchem Zielwert?. Herz.

[54] Gupta A, Mackay J, Whitehouse A (2018). Long-term mortality after blood pressure-lowering and lipid-lowering treatment in patients with hypertension in the Anglo-Scandinavian Cardiac Outcomes Trial (ASCOT) Legacy study: 16-year follow-up results of a randomised factorial trial. Lancet.

